# Supplementary material for: Clinical Implications of the Genetic Background in Pediatric Pulmonary Arterial Hypertension: Data from the Spanish REHIPED Registry
Source: Int J Mol Sci. 2022 Sep 9;23(18):10433. doi: 10.3390/ijms231810433 (PMC9499494; doi:10.3390/ijms231810433)
Supplement: Supplementary file 1 [file ijms-23-10433-s001.zip › Table S1.pdf]

Supplementary Table S1. Description of the genetic findings by clinical group. In silico prediction (pathogenic prediction/benign prediction); LP (likely pathogenic); P (pathogenic); PAH (Pulmonary Arterial Hypertension); PAH-CHD (Pulmonary Arterial Hypertension associated with congenital heart disease); PVOD (pulmonary venooclusive disease); NA: not available.

	Gene name	cDNA	Protein	Type of mutation	Zygosity	In silico prediction+ CADD phred	ACMG
**Idiopathic PAH**							
Chr2:203424566	*BMPR2*	NM_001204.6:c.3014G>A	p.(Ser1005Asn)	Missense	Heterozygous	4/20 + 12.8	VUS
Chr15:40318179	*EIF2AK4*	NM_001013703.4:c.4392dupT	p.(Lys1465Ter)	Nonsense	Heterozygous	. + 37	P
Chr8:55370916	*SOX17*	NM_022454.3:c.218A>C	p.(Asn73Thr)	Missense	Heterozygous	25/0 + 26.7	VUS
Chr4:146435805	*SMAD1*	NM_001003688:c.40G>A	p.(Val14Met)	Missense	Heterozygous	23/1 + 26.6	VUS
Chr13:37427668	*SMAD9*	NM_005905:c.1037A>C	p.(Lys346Thr)	Missense	Heterozygous	24/1 + 23.3	VUS
**Heritable PAH**							
Chr12:52308253	*ACVRL1*	NM_001077401:c.656G>A	p.(Gly219Asp)	Missense	Heterozygous	25/0 + 29	LP
Chr7:30951900	*AQP1*	NM_198098.3:c.376C>T	p.(Arg126Cys)	Missense	Heterozygous	15/7 + 25.9	LP
Chr4:96051161	*BMPR1B*	NM_001256793.1:c.824C>G	p.(Thr275Arg)	Missense	Heterozygous	24/1 + 28.2	LP
NA<br>Chr19:15296478	*BMPR2*<br>*NOTCH3*	Exon 2 duplication<br>NM_000435.2:c.1964A>G	-<br>p.(Asn655Ser)	Duplication<br>Missense	Heterozygous<br>Heterozygous	-<br>15/9 + 25.5	LP<br>VUS
Chr2:203420941<br>Chr18:48581282	*BMPR2*<br>*SMAD4*	NM_001204.7:c.2553_2555delTATinsAA<br>NM_005359:c.586A>G	p.(Phe851LeufsTer8)<br>p.(Ser196Gly)	Frameshift<br>Missense	Heterozygous<br>Heterozygous	. + 17.78<br>8/16 + 21	P<br>VUS
Chr2:203407080	*BMPR2*	NM_001204:c.1325delA	p.(Asn442ThrfsTer32)	Frameshift	Heterozygous	. + 31	P
NA	*BMPR2*	Exon 7 deletion	-	Deletion	Heterozygous	-	LP
NA	*BMPR2*	Exons 1, 2, 3, 4 and 5 deletion	-	Deletion	Heterozygous	-	P
NA	*BMPR2*	Exons 5, 6 and 7 deletion	-	Deletion	Heterozygous	-	P
Chr2:203417484	*BMPR2*	NM_001204:c.1459G>C	p.(Asp487His)	Missense	Heterozygous	25/0 + 29.2	P
Chr2:203417449	*BMPR2*	NM_001204.6:c.1424C>G	p.(Ser475Ter)	Nonsense	Heterozygous	9/1 + 39	P
Chr2:203242245	*BMPR2*	NM_001204.7:c.48G>A	p.(Trp16Ter)	Nonsense	Heterozygous	6/3 + 34	P
Chr2:203420888<br>Chr19:15288766	*BMPR2*<br>*NOTCH3*	NM_001204.6:c.2500C>T<br>NM_000435.2:c.3973C>T	p.(Gln834Ter)<br>p.(Arg1325Cys)	Nonsense<br>Missense	Heterozygous<br>Heterozygous	9/1 + 38<br>16/7 + 24.3	P<br>VUS
Chr10:48416366	*GDF2*	NM_016204.3:c.328C>T	p.(Arg110Trp)	Missense	Homozygous	1/0 + 32	P
Chr2:26950567	*KCNK3*	NM_002246.2:c.316G>C	p.(Gly106Arg)	Missense	Homozygous	21/4 + 27.5	LP
Chr2:26950567	*KCNK3*	NM_002246.2:c.316G>C	p.(Gly106Arg)	Missense	Heterozygous	21/4 + 27.5	LP
**PAH-CHD**							
Chr11:17496512<br>Chr4:146435792	*ABCC8*<br>*SMAD1*	NM_000352.4:c.211C>T<br>NM_005900.2:c.27delinsGTAAAG	p.(His71Tyr)<br>p.(Phe9LeufsTer2)	Missense<br>Frameshift	Heterozygous<br>Heterozygous	25/0 + 26.1<br>. + 29.9	LP<br>LP
Chr2:203420986	*BMPR2*	NM_001204.6:c.2598G>C	p.(Glu866Asp)	Missense	Heterozygous	16/8 + 22.9	VUS
NA	BMPR2	Exons 3, 4, 5, 6 y 7 deletion	-	Deletion	Heterozygous	-	P
Chr2:203383726	*BMPR2*	NM_001204:c.803C>G	p.Thr2685Ser	Missense	Heterozygous	8/14 + 20.9	VUS
Chr2:211457683	*CPS1*	NM_001875.5:c.1164+3_1164+4delAC	-	Deletion	Heterozygous	. + 23.6	VUS
Chr9:130605513	*ENG*	NM_000118.2:c.79A>G	p.(Thr27Ala)	Missense	Heterozygous	0/24 + 16.74	VUS
Chr8:55370942	*SOX17*	NM_022454:c.244G>T	p.(Glu82Ter)	Nonsense	Heterozygous	9/1 + 40	P
**PVOD**							
Chr15:40295502	*EIF2AK4*	NM_001013703.3:c.3344C>T	p.(Pro115Leu)	Missense	Homozygous	20/5 + 32	P
Chr15:40295502	*EIF2AK4*	NM_001013703.3:c.3344C>T	p.(Pro115Leu)	Missense	Homozygous	20/5 + 32	P
Chr15:40295502	*EIF2AK4*	NM_001013703.3:c.3344C>T	p.(Pro115Leu)	Missense	Homozygous	20/5 + 32	P

Chr15:40246149	*EIF2AK4*	NM_001013703.3:c.558_562delAAAAG	p.(Lys187fsTer9)	Nonsense	Homozygous	-	P
Chr15:40265885<br>Chr15:40293255	*EIF2AK4*<br>*EIF2AK4*	NM_001013703.4:c.1753C>T<br>NM_001013703.4:c.2989_2990delAA	p.(Arg585Ter)<br>p.(Lys997GlufsTer2)	Nonsense<br>Frameshift	Compound heterozygous	9/1 + 38<br>. + 35	P<br>P
Chr15:40295502	*EIF2AK4*	NM_001013703.3:c.3344C>T	p.(Pro115Leu)	Missense	Homozygous	20/5 + 32	P
Chr15:40295502	*EIF2AK4*	NM_001013703.3:c.3344C>T	p.(Pro1115Leu)	Missense	Homozygous	20/5 + 32	P
Chr15:40295502	*EIF2AK4*	NM_001013703.3:c.3344C>T	p.(Pro1115Leu)	Missense	Homozygous	20/5 + 32	P
**TBX4 mutations**							
Chr17:59557677	TBX4	NM_018488.3:c.1018C>T	p.(Arg340Ter)	Nonsense	Heterozygous	9/1 + 39	P
Chr17:58113570-60325222	*TBX4*	Complete deletion	-	Deletion	Heterozygous	-	VUS
Chr17:58113570-60325222	*TBX4*	Complete deletion	-	Deletion	Heterozygous	-	VUS
Chr17:58113570-60325222	*TBX4*	Complete deletion	-	Deletion	Heterozygous	-	VUS
**Developmental lung disorders**							
NA	*FOXF1*	Complete deletion	-	Deletion	Homozygous	-	LP
Chr16:86544432	*FOXF1*	NM_001451.2:c.257G>C	p.(Arg86Pro)	Missense	Heterozygous	23/0 + 32	P
**Multisystemic disorders**							
Chr3:81627227	*GBE1*	NM_000158.4:c.1467delC	p.(Leu490TrpfsTer5)	Frameshift	Heterozygous	-	LP
Chr2:69627594	*NFU1*	NM_001002755.4:c.622G>T	p.(Gly208Cys)	Missense	Homozygous	24/0 + 29.5	P
Chr2:69627594	*NFU1*	NM_001002755.4:c.622G>T	p.(Gly208Cys)	Missense	Homozygous	24/0 + 29.5	P
Chr2:69627594	*NFU1*	NM_001002755.4:c.622G>T	p.(Gly208Cys)	Missense	Homozygous	24/0 + 29.5	P
Chr2:69627594	*NFU1*	NM_001002755.4:c.622G>T	p.(Gly208Cys)	Missense	Homozygous	24/0 + 29.5	P
Chr1:45973021	*MMACHC*	NM_015506.2:c.82-7_82-5delGTT	-	Deletion	Homozygous	-	LP
ChrX:153296363	*MECP2*	NM_001110792.2:c.952C>T	p.(Arg318Cys)	Missense	Heterozygous	8/3 + 32	P
NA	*MECP2*	Complete deletion	-	Deletion	Heterozygous	-	P
ChrX:149116213-154926279	*MECP2*	Complete duplication	-	Duplication	Heterozygous	-	P
Chr3:10188233	*VHL*	NM_000551.4:c.376G>A	p.(Asp126Asn)	Missense	Heterozygous	19/2 + 25.2	P
