# Supplementary material for: Clinical Implications of the Genetic Background in Pediatric Pulmonary Arterial Hypertension: Data from the Spanish REHIPED Registry
Source: Int J Mol Sci. 2022 Sep 9;23(18):10433. doi: 10.3390/ijms231810433 (PMC9499494; doi:10.3390/ijms231810433)
Supplement: Supplementary file 1 [file ijms-23-10433-s001.zip › Table S2.pdf]

Supplementary Table S2. Description of the clinical, analytical, hemodynamical and follow-up data of patients with pulmonary hypertension associated with *TBX4* variants. ASD (atrial septal defect), CI (cardiac index); C (Caucasian); CCB (calcium channel blockers); CO (cardiac output); F (female); H (Hispanic); ILD (interstitial lung disease); M (male); LP (likely pathogenic); mPAP (mean pulmonary artery pressure); PAWP (pulmonary artery wedge pressure); PH (pulmonary hypertension); PDA (patent ductus arteriosus), PFO (patent foramen ovale); PVR (pulmonary vascular resistance); RAP (right atrial pressure); WU (Wood Units).

Case	Associated conditions	Age at diagnosis	Sex	Race	RHC	Therapy	Pathologic findings	Genetic findings
1	Small sinus venosus defect and partial anomalous pulmonar and venous connection.<br><br>Mild intellectual disability	15 years	M	C	mPAP 61 mmHg, PVR 16.5 WU*m2	Triple sequential therapy with sc. treprostinil	Alive	*TBX4* (P)<br><br>NM_018488.3:c.1018C>T: p.(Arg340*)
2	Mild intellectual disability	2 months age	F	C	mPAP 27 mmHg. PVR/SVR 0,79	Double oral sequential therapy (CCB and ERA)	Alive	*TBX4*<br><br>Complete deletion
3	Mild intellectual disability, PFO, small PDA, Lung CT compatible with ILD, oxygen dependance in the first year of age.	Neonatal period	F	C	mPAP 39 mmHg, PAWP 12 mmHg, PVR 5,6 WU*m2.	Double oral sequential therapy (iPDE5 and ERA)	Alive	*TBX4*<br><br>Complete deletion
4	Dysmorphic facial features, Small ASD, oxygen dependance, hypoacusia, neurodevelopmental delay	Neonatal period	M	C	PVR 16.0 WU*m2 (supra-systemic PA pressure)	Double oral sequential therapy (iPDE5 and ERA)	Alive	*TBX4*<br><br>Deletion 17q23, including complete deletion of the TBX4 gene
