# Supplementary material for: Clinical Implications of the Genetic Background in Pediatric Pulmonary Arterial Hypertension: Data from the Spanish REHIPED Registry
Source: Int J Mol Sci. 2022 Sep 9;23(18):10433. doi: 10.3390/ijms231810433 (PMC9499494; doi:10.3390/ijms231810433)
Supplement: Supplementary file 1 [file ijms-23-10433-s001.zip › Table S3.pdf]

Supplementary Table S3. Description of the clinical, analytical, hemodynamical and follow-up data of patients with PAH associated with congenital heart disease. ASD (atrial septal defect), ASO (atrial swithc operation), AV (atrioventricular canal), B (black), C (caucasian), CHD (congenital heart disease), F (female), H (Hispanic), LP (likely pathogenic), LV (left ventricle), M (male), P (pathogenic), PDA (patent ductus arteriosus), PAH (pulmonary arterial hypertension), TGA (transposition of great arteries), VSD (ventricular septal defect), VUS (variant of unknown significance).

**Patient**	**Type of CHD**	**CHD**	**Age at diagnosis**	**Sex**	**Race**	**mPAP**	**RAP**	**CO/CI**	**PVR**	**TAPSE**	**ntproBNP/BNP**	**6MWT**	**Therapy**	**Vital status**	**Comorbidity**	**Genetic findings**
1	Eisenmenger	ASD and PAD	2	F	C					13			Double oral	Death	Autism	7p22.1 duplication
2	PAH-CHD with incidental defects	ASD	18	F	H	57	4	5.4/-	9.4	15	104/-	370 (delta 0%)	Double oral sequential	Alive		*GDF2* (VUS)
3	PAH-CHD with incidental defects	AV canal with incidental ASD and VSD	2	F	C	63	11	-/ 2.1	40.3	15	-/62		Sequential triple (sc Treprostinil)	Alive	Down syndrome	Trisomy 21
4	Eisenmenger	AV canal	18	F	C					31	-/-	330 (delta 24%)	Double oral sequential	Alive	Down syndrome	Trisomy 21
5	Eisenmenger	PDA	13	F	C					20	107/-	440 (delta 4%)	Double sequential with iPDE5 and sc Treprostinil	Alive	Intelectual disability	Duplication in Chromosome 4q35
6	PAH after defect closure	Corrected D-TGA (ASO)	7	M	C	34	7	5.3/6.5	4.3	11	618/-	528 (1%)	Double initial oral therapy	Alive		
7	PAH after defect closure	Corrected PDA	1	F	C	118	11	-/3.0	36	20	-/40	528 (delta 1%)	Triple initial therapy with sc Treprostinil	Alive		

8	PAH-CHD with incidental defects	Incidental ASD and VSD	10	M	H	80	7	-/6.0	23.3	26	-/62	600 (delta 29%)	Triple sequential therapy with sc Treprostinil	Alive		*SMAD1* (LP)/ *ABCC8* (LP)
9	PAH after defect closure	ASD and left atrial isomerism	9	F	C	43	10	-/6.7	5.4	9	-/35	384 (delta 0%)	Triple sequential therapy with sc Treprostinil	Alive		*CPS1* (VUS)
10	Eisenmenger	AV canal	1	F	C					25	-/93	333 (delta 10%)	Double oral sequential	Alive	Down syndrome, prematurity	*BMPR2* (VUS) Trisomy 21
11	PAH after defect closure	Corrected PDA	3	F	C	52	6	-/4.0	7.5	17	-/90	420 (delta 18%)	Triple sequential therapy with sc Treprostinil	Alive	Broncopulmonary dysplasia, prematurity	
12	PAH after defect closure	Corrected D-TGA (ASO)	9	M	C	65	10	-/1.7	30.0	17	-/25	670 (delta 4%)	Triple sequential therapy with sc Treprostinil	Alive		*ENG* (VUS)
13	PAH with congenital systemic-pulmonary shunts	ASD	7	F	C	63	6	-/4.2	13.1	22	845/-		Double oral sequential	Alive	Prematurity, Intelectual disability	
14	PAH with congenital systemic-pulmonary shunts	Small ASD, small muscular VSD and ample PDA	0	F	H	31	6	-/6.0	1.6	20	25431/-		Triple sequential therapy with sc Treprostinil	Alive		
15	PAH after defect closure	Corrected D-TGA (ASO)	4	M	C					14	328/-	335 (delta 4%)	Double oral sequential	Alive		*BMPR2* (VUS)
16	PAH after defect closure	Corrected VSD	4	F	C	48	8	-/1.7	22.6	15	-/-	345 (delta 3%)	Double oral sequential	Alive	Prematurity	
17	PAH after defect closure	Corrected D-TGA (ASO)	5	F	C	63	8	-/3.1	17.4	12	141/-	390 (delta 8%)	Triple sequential therapy with sc Treprostinil	Alive	Potts shunt at 11 year	
18	PAH after defect closure	Corrected VSD	3	M	C	77	17	-/4.0	18.3	11	-/-	247 (delta 5%)	Triple sequential therapy with inhaled iloprost	Alive		*BMPR2* (P)
19	PAH after defect closure	Corrected PDA	14	F	C	77	6	6.2/-	10.3	22	208/-	450 (delta 4%)	Double oral sequential	Alive		

20	PAH after defect closure	Corrected PDA	1	F	C				4.5				Double oral sequential	Alive	Broncopulmonary dysplasia, prematurity	
21	PAH after defect closure	Corrected D-TGA (Mustard)	18	M	C	62	4	-/4.8	12.0	16	-/43	768 (delta 4%)	Double oral sequential	Alive		
22	PAH with congenital systemic-pulmonary shunts	Big ASD	8	M	B	61	10	-/3.8	11.8	12	-/31	440 (delta 12%)	Triple sequential therapy with sc Treprostinil	Alive	Chronic total occlusion of the left main coronary artery due to PA aneurism	
23	PAH after defect closure	AV canal and aortic coarctation	12	F	C				13.0	13	-/55	528 (delta 7%)	Triple sequential oral therapy	Alive	Down syndrome/ Prematurity	Trisomy 21
24	Eisenmenger	Double inlet LV, D-TGA and PAD	2	M	C	40	9		2.3		512/-	198 (delta 9%)	Double oral sequential	Alive		
25	PAH with congenital systemic-pulmonary shunts	Sinus venosus defect and partial anomalous pulmonary venous connection	14	F	C	53	5	5.3/3.3	8.5		923/-	370 (delta 8%)	Triple sequential therapy with sc Treprostinil	Alive		
26	PAH after defect closure	Corrected D-TGA (ASO)	8	M	C	85	7	5.5/4.4	17.0	11	440/-	593	Triple sequential therapy with sc Treprostinil	Alive		
27	Eisenmenger	Left atrial isomerism, ASD and partial anomalous	1	M	C	44			4.6				Double oral sequential	Alive		*SOX17* (P)

		venous drainage														
28	PAH after defect closure	Corrected D-TGA (ASO)	1	M	C	46	10	4.0	9.1				Double oral sequential	Alive		
