# Supplementary material for: Clinical Implications of the Genetic Background in Pediatric Pulmonary Arterial Hypertension: Data from the Spanish REHIPED Registry
Source: Int J Mol Sci. 2022 Sep 9;23(18):10433. doi: 10.3390/ijms231810433 (PMC9499494; doi:10.3390/ijms231810433)
Supplement: Supplementary file 1 [file ijms-23-10433-s001.zip › Table S4.pdf]

Supplementary Table S4. Description of the clinical, analytical, hemodynamical and follow-up data of patients with pulmonary hypertension in multisystemic disorders. A (Arabic), C (Caucasian); CO (cardiac output); ERA (endothelin receptor antagonists); F (female); H (Hispanic); M (male); mPAP (mean pulmonary artery pressure); N/A (not available); PAWP (pulmonary artery wedge pressure); PA (pulmonary artery); PH (pulmonary hypetension; PVR (pulmonary vascular resistance); SPAP (systolic pulmonary artery pressure), TTE (transthoracic echocardiography), WU (Wood Units).

Patient	Perinatal history	Associated conditions	Age at diagnosis	Sex	Race	RHC	Therapy	Pathologic findings	Genetic findings
**1**	Intrauterine growth restriction type I<br><br>Full term (37 weeks)<br><br>1.94 Kg at birth	Big ASD, Neonatal Hypotonia, Congenital Aural Stenosis, Congenital Cataracts and Neonatal Apnea.	6 months	M	C	mPAP 43 mmHg<br>RAP 7 mmHg<br>PAWP 8 mmHg<br>CI 2.3 l/m/m2<br>PVR 7 WU*m2	None. Supportive	PVOD	Heterozygous *GBE1* (LP)<br><br>*GBE1*:NM_000158.4:c.1467delC;p.Leu490TrpfsTer5
**2**	Post term (42 weeks)<br><br>3.87 Kg at birth		2 months	F	H	PVR 22.0 WU*m2	Inhaled nitric oxide, sildenafil and bosentan	N/A	Homozygous *NFU1* (P)<br><br>*NFU1*:NM_001002755.4:c.622G>T: p.Gly208Cys
**3**	Full term (37 weeks)<br><br>3.71 Kg at birth	Hypothyroidism	5 months	M	C	mPAP 46 mmHg<br>RAP 14 mmHg<br>PAWP 10 mmHg<br>CO 2.5 l/m<br>PVR 14.3 WU*m2	Sildenafil, bosentan and treprostinil	N/A	Homozygous *NFU1* (P)<br><br>*NFU1*:NM_001002755.4:c.622G>T: p.Gly208Cys
**4**	Full term (38 weeks)<br><br>3.30 Kg at birth		1 month	F	A	N/A (TTE with estimated SPAP of 110 mmHg and TAPSE of 15 mm)	Sildenafil and epoprostenol	N/A	Homozygous *NFU1* (P)<br><br>*NFU1*:NM_001002755.4:c.622G>T: p.Gly208Cys

**5**	Full term (37 weeks)		2 months	F	C	mPAP 36 mmHg<br>RAP 6 mmHg<br>PAWP 7 mmHg<br>CI 3.9 l/m/m2<br>PVR 7.5 WU*m2	Double oral initial	N/A	Homozygous *NFU1* (P)<br><br>*NFU1*:c.550G>T: p.Gly184Cys
**6**	Full term	Initial clinical suspicion of PVOD by means of a DLCO of 30% and radiological findings of probable PVOD.<br><br>Hemolytic uremic syndrome and associated end-stage kidney disease, neurogenic amyotrophy of the lower limbs, and hyperhomocysteinemia.<br><br>Correction of PH after kidney transplantation and hydroxocobalamin, betaine, carnitine, and folic acid supplementation	16 years	M	C	mPAP 53 mmHg<br>RAP 10 mmHg<br>PAWP 7 mmHg<br>CO 6.5 l/m<br>PVR 7.1 WU*m2	Sildenafil	Alive	Cobalamin C deficiency. *MMACHC* (LP)<br><br>*MMACHC*_015505.2:c.82_7_825delGTT
**7**	Full term	Alveolar capillary dysplasia with misalignment of the pulmonary veins associated with aortic coarctation and intestinal malrotation	1 month	M	C	Supra-systemic PA pressure	ECMO	Congenital alveolar dysplasia, misalignment of pulmonary veins, arteriolar vascular lesions consisting of PAH	1.65-Mb de novo hemizygous deletion of chromosome 16q24.1-q24. This deletion contained four annotated genes (*FOXF1*, *FOXC2*, *FOXL1* and *JPH3*)
**8**	Full term	Alveolar capillary dysplasia, partial AV canal, PH, and intestinal malrotation	1 month	M	C	mPAP 42 mmHg,<br>PAWP 7 mmHg,<br>PVR 6.5 WU*m2 (130% of systemic resistances)	Nitric oxide<br>Sildenafil<br>Bosentan<br>Suportive	Congenital alveolar dysplasia	*FOXF1* (P)<br><br>*FOXF1*:NM_001451.2:c.257G>C:p.Arg86Pro

**9**	Prematurity (33 weeks)<br><br>1.8 Kg at birth		1 month	F	C	PVR 10.7 WU*m2	Double oral initial therapy with iPDE5 and ERA	N/A	MECP2 (LP)<br><br>*MECP2*:NM_001110792.2:c.952C>T:p.Arg318Cys
**10**	Full term: 42 weeks<br><br>2.7 Kg at birth	Neonatal Hypotonia, neonatal hypoglycemia, deafness, psychomotor retardation, neonatal bone disorders	13 months	M	C	N/A (TTE with estimated SPAP of 110 mmHg and TAPSE of 5 mm). ntproBNP of 28486 ng/dL	Triple sequential therapy with treprostinil	N/A	Xq28 microduplication, including *MECP2* gene
**11**	Full term: 40 weeks<br><br>3.3 Kg at birth	Neonatal Hypotonia, psychomotor retardation	1 month	F	C	mPAP 27 mmHG<br>RAP 15 mmHg<br>PAWP 15 mmHg<br>CI 2.0 l/min/m2<br>PVR 27.0 WU*m2	Triple sequential therapy with inhaled iloprost	Obstructive vasculopathy with plexiform lesions, subintimal proliferation and thrombosis. Septal venous affection (PVOD-like lesions)	Xq28 duplication, including *MECP2*, *GDI1* and *RAB39B* genes. Yq associated deletion.
**12**	Full term: 39 weeks	retinal arteriolar *tortuosity* and *erythrocytosis*	3 months	M	A	mPAP 39 mmHg<br>RAP 6 mmHg<br>PAWP 7 mmHg<br>PVR 3.7 WU*m2	Double sequential therapy with inhaled iloprost and ERA	Lost after 10 years of follow-up	*VHL* (P)<br><br>*VHL*:NM_000551.4: c.376G>A: p.Asp126Asn
