# Supplementary figures and images for: Clinical Implications of the Genetic Background in Pediatric Pulmonary Arterial Hypertension: Data from the Spanish REHIPED Registry
Source: Int J Mol Sci. 2022 Sep 9;23(18):10433. doi: 10.3390/ijms231810433 (PMC9499494; doi:10.3390/ijms231810433)

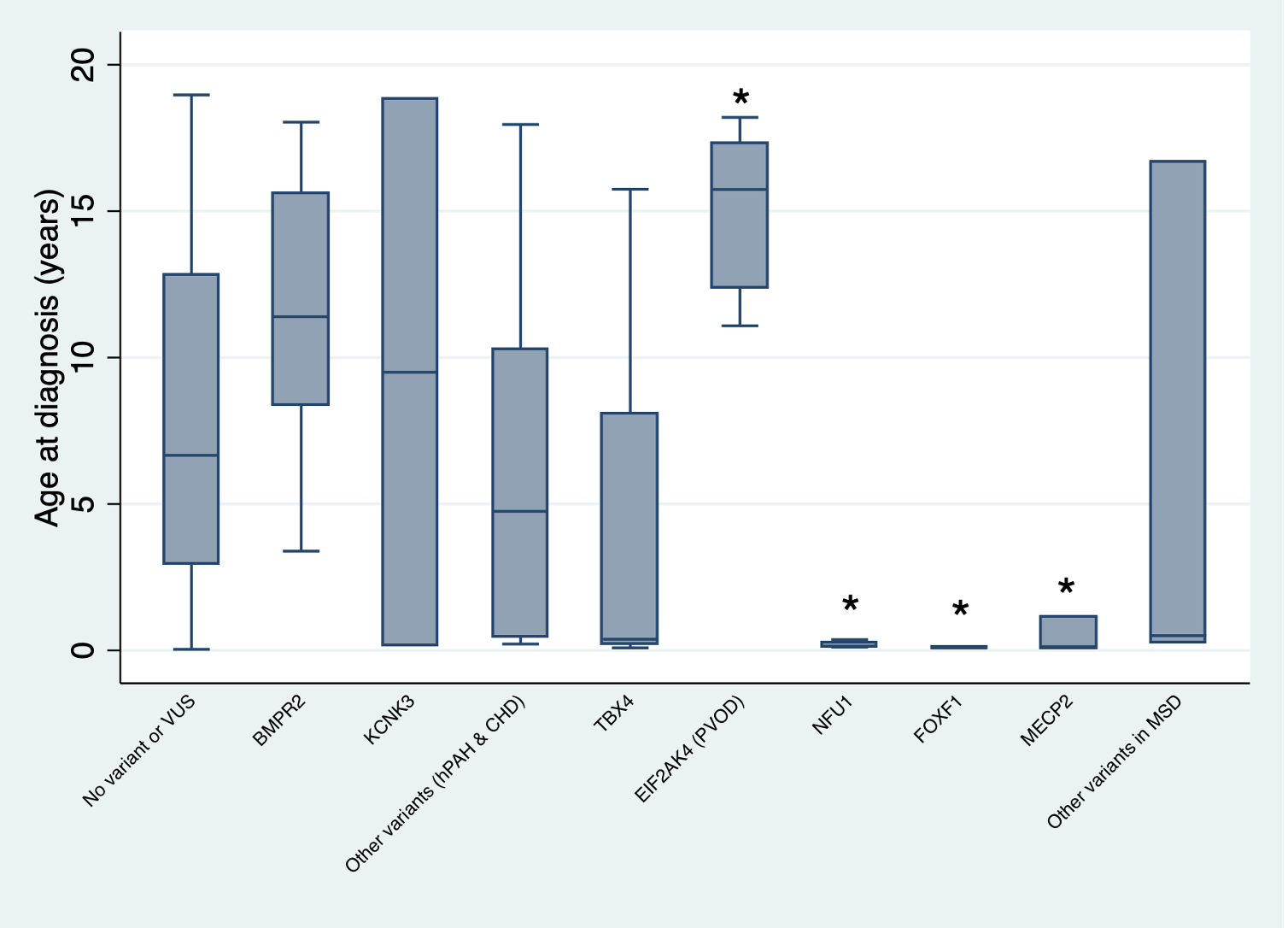

Supplement: Supplementary file 1 [file ijms-23-10433-s001.zip › Figure S1.png]
